# Climate factors associated with the population dynamics of *Sitodiplosis mosellana* (Diptera: Cecidomyiidae) in central China

**DOI:** 10.1038/s41598-019-48868-w

**Published:** 2019-08-26

**Authors:** Jin Miao, Jianrong Huang, Yuqing Wu, Zhongjun Gong, Huiling Li, Guoyan Zhang, Yun Duan, Tong Li, Yueli Jiang

**Affiliations:** 1 Institute of Plant Protection, Henan Academy of Agricultural Sciences, Key Laboratory of Crop Pest Control of Henan Province, Key Laboratory of Pest Management in South of North-China, Ministry of Agriculture, Zhengzhou, 450002 China; 2 Plant Protection and Plant Quarantine Station of Henan Province, Zhengzhou, 450002 China

**Keywords:** Behavioural ecology, Entomology

## Abstract

Understanding the impacts of climate on insect pest population dynamics is crucial in forecasting pest outbreaks and developing a sustainable pest management strategy. The orange wheat blossom midge, *Sitodiplosis mosellana* (Géhin), is a chronic winter wheat (*Triticum aestivum* L.) pest in China, and its population density can strongly fluctuate. We analyzed climate factors (temperature and precipitation) associated with population dynamics of *S. mosellana* in a large-scale field trial in central China from 1984 to 2013 using Generalized linear mixed effects models. We found total precipitation during January–March was significantly positively correlated with population density of *S. mosellana*, whereas temperature parameters were not correlated with the population levels. Moreover, *S. mosellana* population size was significantly negative effected by interaction between temperature and precipitation, which showed that high precipitation with low temperature in spring also reduced the population density. This suggests that annual population size of *S. mosellana* in Central China is determined by soil moisture in early spring. These results provide basic information that will help in forecasting population levels and in developing a sound pest management strategy for *S. mosellana*.

## Introduction

Weather influences pest population dynamics directly or indirectly by affecting survival, behavior, and life cycles^1^. The geographical distribution and phenology of many major crop insect pest species have been affected by global climate change. For example, high temperatures increase the hatch rate and development of overwintering *Apolygus lucorum* (Meyer-Dür) eggs^2^. A temperature increase of 2 °C can add two generations of *Plutella xylostella* (L.) and five generations of *Myzus persicae* (Sulzer) per year in a large population^3,4^. Moisture factors such as precipitation and humidity may also influence population growth and seasonal dynamics of *A. lucorum*^2^ and *Mythimna separata* (Walker)^5^. However, insect population dynamics are often affected by multiple climate factors. Scherber *et al*. found that ambient CO_2_, temperature, and drought affected population size and growth of *Lochmaea suturalis* (Thomson) by acting on larval weight and survival^6^.

The orange wheat blossom midge, *Sitodiplosis mosellana* (Géhin) (Diptera: Cecidomyiidae), is a chronic wheat (*Triticum aestivum* L.) pest found throughout the northern hemisphere. This species is univoltine, and larvae feed on the developing wheat kernels, which considerably reduces grain yield and quality^7,8^. *S. mosellana* has been an important pest of winter wheat in some regions of China since the 1950s, and population levels often experience strong year-to-year fluctuations. Two disastrous outbreaks of *S. mosellana* over large areas of China were recorded in 1951 and 1989, causing substantial reductions in wheat yields^9,10^.

The diapause larvae of *S. mosellana* overwinter in cocoons within the soil and suffer losses ranging from 0 to 81% during the winter, which cannot be predicted^11^. The diapause larvae require exposure to cold temperatures (−5 to −2 °C) for at least 3 months^12^. Development begins in response to rising soil temperatures, each spring the proportion of the total population that develops and pupates in a given year is determined by soil temperature and moisture^13^. If conditions are moist, larvae move to the soil surface to pupate, whereas if dry, larvae remain in extended diapause for at least one additional year^14^. Adults emerge and females oviposit on the spikes of wheat and hatch after 4–10 days. Larvae feed on the developing kernels for 3–4 weeks, and mature larvae drop to the soil surface and enter the soil to overwinter^15^.

The populations of *S. mosellana* fluctuate yearly at the local level^15^ but the key factors determining population levels of *S. mosellana* remain uncertain. Many studies have investigated the key factors driving population dynamics of other insect pests and their natural enemies for the purposes of forecasting future outbreaks^2,15^. However, most studies were not conducted for extended time periods or over large spatial scales. Our research focused on determining the key climate factors (temperature and precipitation) influencing population dynamics of *S. mosellana* in Henan province, central China. We collected data over a 30-year period (1984–2013).

## Results

### Climate change and population dynamics of *S. mosellana*

**Figure 1:**
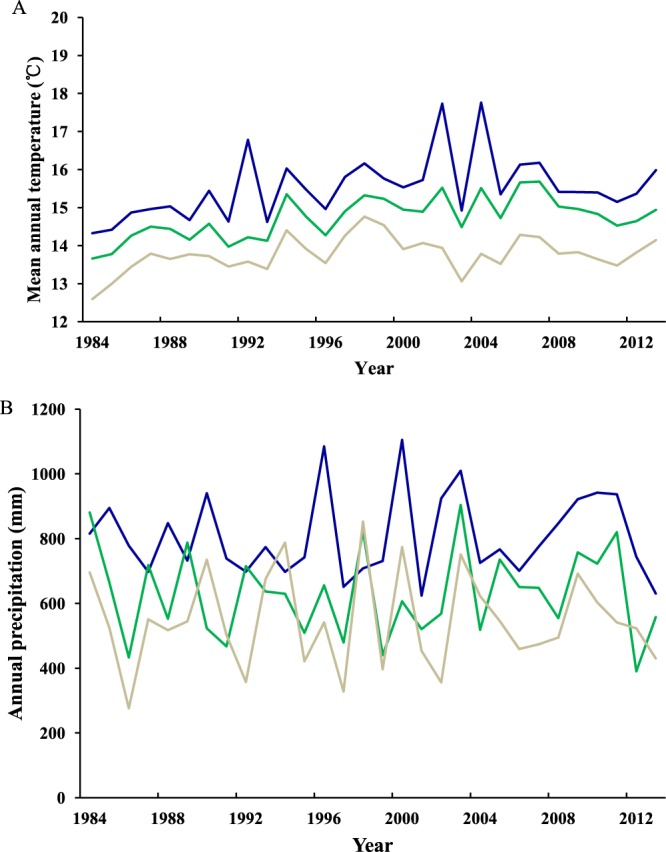
Annual mean temperature (**A**) and annual total precipitation (**B**) in the southern regions (blue line), central regions (green line), and northern regions (beige line) of Henan Province during 1984–2013.

From 1984 to 2013, the annual mean temperature and annual total precipitation varied significantly among years in the southern, central, and northern regions of Henan province (Temperature: southern region, *F*_29, 148_ = 4134.74, *P* < 0.001; central region, *F*_29, 148_ = 1885.33, *P* < 0.001; northern region, *F*_29, 148_ = 2594.62, *P* < 0.001; Precipitation: southern region, *F*_29, 148_ = 9524.43, *P* < 0.001; central region, *F*_29, 148_ = 8628.96, *P* < 0.001; northern region, *F*_29, 148_ = 12755.41, *P* < 0.001; Fig. 1).

**Figure 2:**
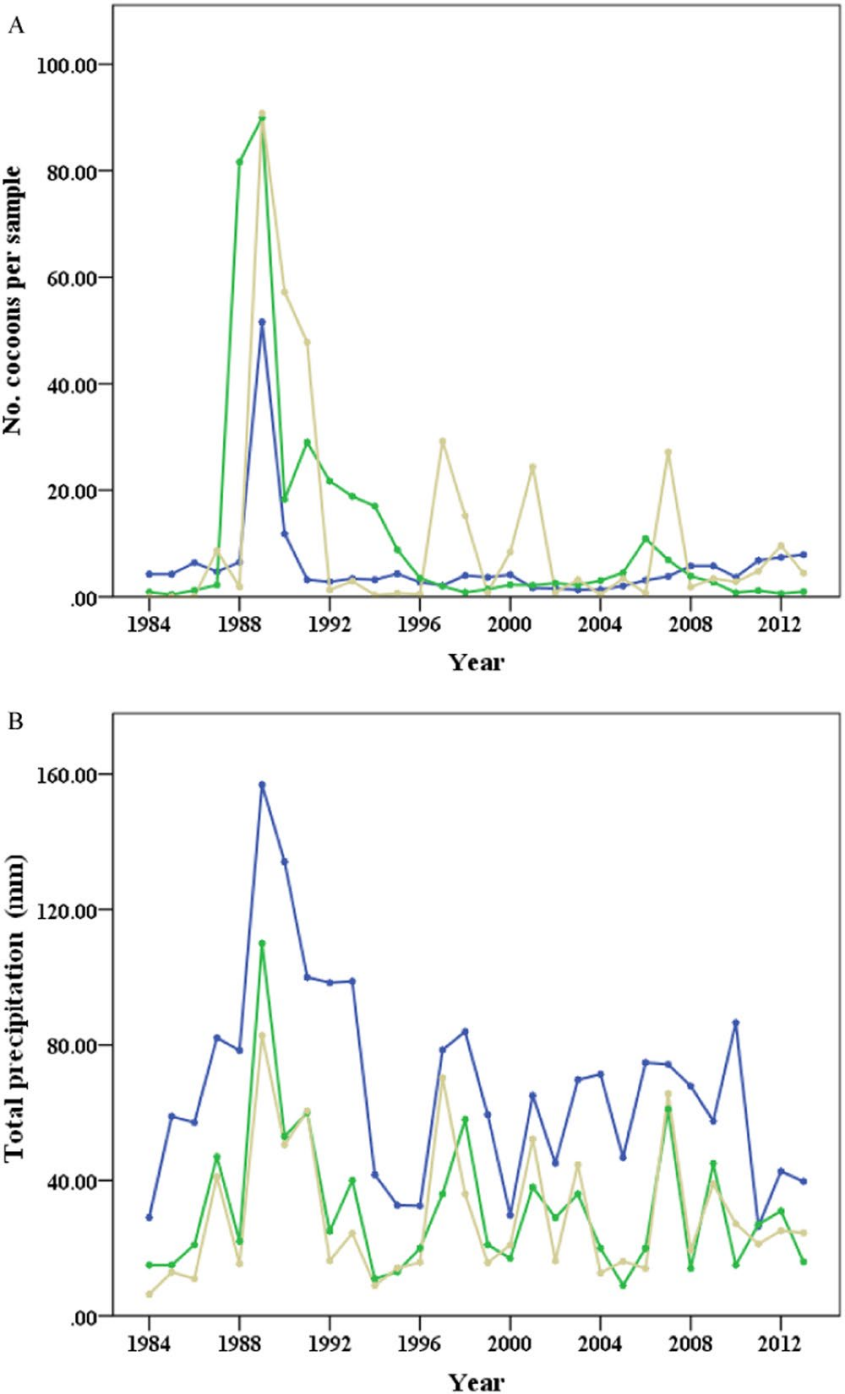
Population density (cocoon) of *S. mosellana* (**A**) and total precipitation from January to March (**B**) in southern regions (blue line), central regions (green line), and northern regions (beige line) of Henan Province during 1984–2013.

The annual population densities of *S. mosellana* varied significantly within each region without a clear trend (southern region, *F*_29, 148_ = 28730.44, *P* < 0.001; central region, *F*_29, 148_ = 416793.35, *P* < 0.001; northern region, *F*_29, 148_ = 814126.58, *P* < 0.001). All regions had a population outbreak in 1989. No significant differences were observed between regions in the population levels of *S. mosellana* over 30 years (*F*_2, 498_ = 0.801, *P* = 0.495; Fig. 2).

### Temperature and precipitation variables

Mean temperature were not significantly correlated with the monthly abundance of *S. mosellana* in all three regions. The population dynamics of *S. mosellana* was positively related to monthly precipitation in all regions. However, the interaction between temperature and precipitation was negatively related to monthly abundance in all three regions (Table 1).

**Table 1:** Results of generalized linear mixed effects models relating the population dynamics of *S. mosellana* in southern regions, central regions and northern regions of Henan Province to explanatory variables during 1983–2014.

Explanatory variables	dDF	F- value	P- value
**Southern Region**			
(Intercept)	320	3.318152	0.0014**
temperature	7	0.938323	0.3487
precipitation	7	2.170126	0.0307*
temperature × precipitation	7	−2.283567	0.0230*
**Central Region**			
(Intercept)	320	3.522008	0.0005***
temperature	7	−0.551249	0.5986
precipitation	7	2.64468	0.0179*
temperature × precipitation	7	−2.91417	0.0068**
**Northern Region**			
(Intercept)	320	3.639	0.0004***
temperature	7	0.671	0.50256
precipitation	7	2.891	0.0073**
temperature × precipitation	7	−2.7114	0.00947**

Note: “year” is random variable. ***P < 0.001, **P < 0.01, *P < 0.05.

In Table 2, further analysis shows that total precipitation during January–March was the significant positive predictors of annual variation in population dynamics of *S. mosellana* (southern and northern, *P* < 0.01; central regions, *P* < 0.05). There was a significantly negative role of interaction between temperature and precipitation for *S. mosellana* (southern and northern, *P* < 0.01; central regions, *P* < 0.05).

**Table 2:** Results of generalized linear models relating the population dynamics of *S. mosellana* to explanatory variables during January to March in Henan Province.

Explanatory variables	Estimate	SE	t value	Pr(>|t|)
**Southern Region**				
(Intercept)	−37.7332	15.4248	−2.446	0.02151*
temperature	6.7617	2.9062	0.327	0. 8042
precipitation	2.5062	0.692	3.622	0.00124**
temperature × precipitation	−0.4063	0.133	−3.054	0.00515**
**Central Region**				
(Intercept)	4.184208	1.261005	3.718	0.0010***
temperature	0.078071	0.083202	0.938	0.3487
precipitation	0.070507	0.03249	2.17	0.0307*
temperature × precipitation	−0.003059	0.00134	−2.284	0.0230*
**Northern Region**				
(Intercept)	8.8039	2.56585	5.817	<4e-7***
temperature	0.08717	0.0282	0.425	0.691
precipitation	0.10797	0.09496	2.836	0.00875**
temperature × precipitation	−0.009622	0.04773	−3.623	0.00124**

Note: ***P < 0.001, **P < 0.01, *P < 0.05.

**Figure 3:**
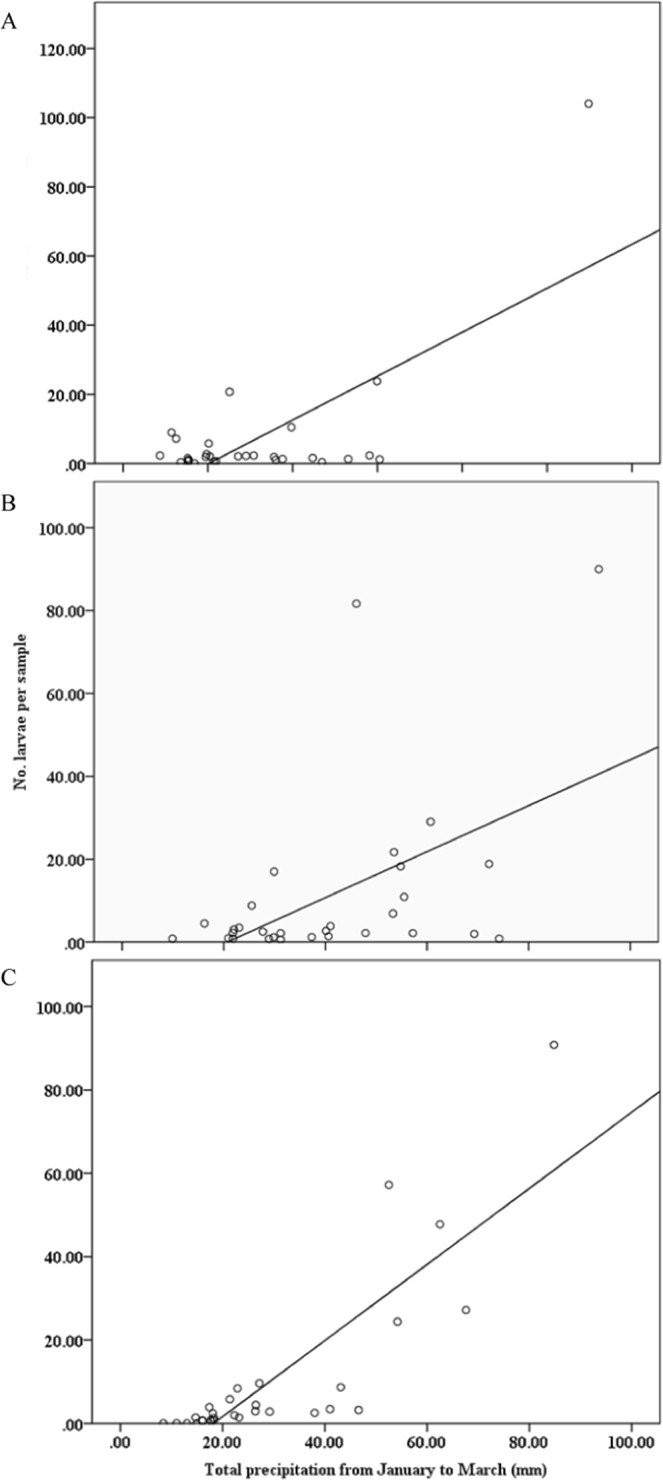
Relationship between total precipitation from January to March and population density (cocoon) of *S. mosellana* in the southern regions (**A**): *y* = 0.15*x* − 4.89, *R*^2^ = 0.299, *P* = 0.003), central regions (**B**): *y* = 0.68*x* − 17.74, *R*^2^ = 0.467, *P* < 0.001), and northern regions (**C**): *y* = 0.85*x* − 13.28, *R*^2^ = 0.701, *P* < 0.001) of Henan Province during 1984–2013.

**Figure 4:**
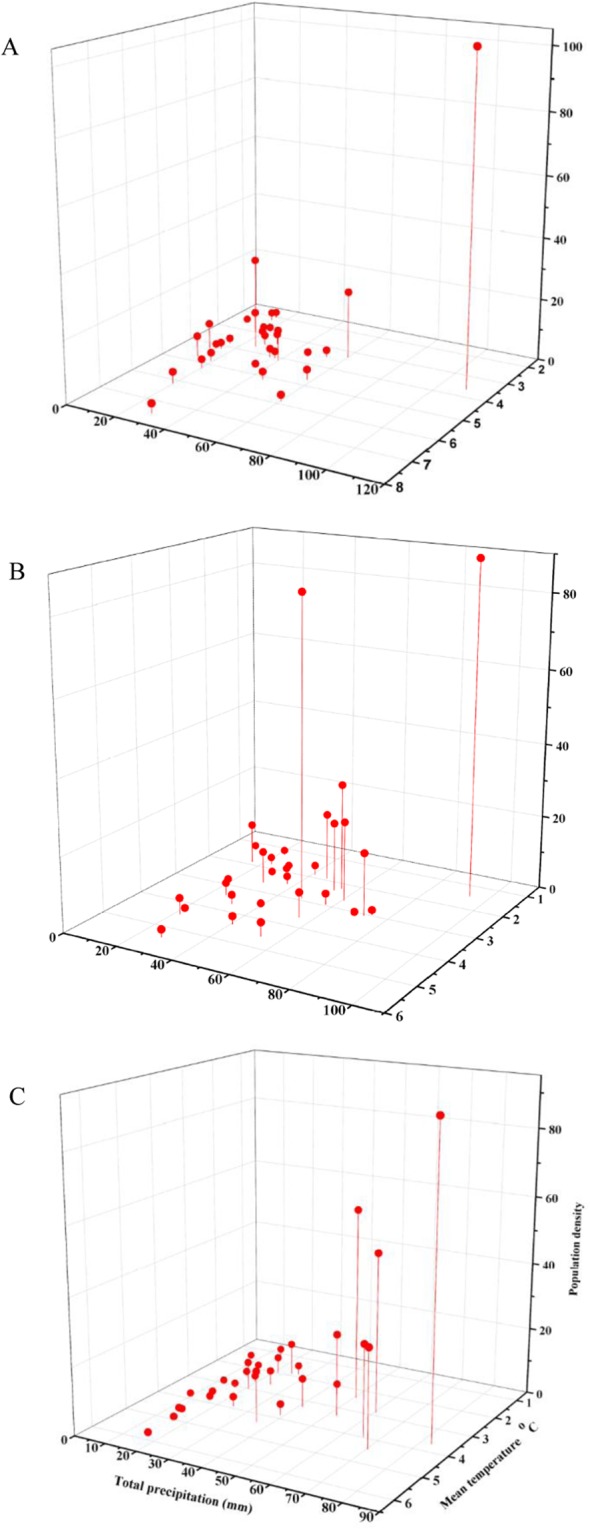
The interaction between precipitation and temperature during January to March on population density in the southern regions (**A**), central regions (**B**), and northern regions (**C**).

The population densities of *S. mosellana* increased linearly with an increase of precipitation during January–March. The linear model for population density in all regions indicated that field population size was generally low if precipitation during January–March was less than about 20 mm (Fig. 3). The interaction between precipitation and temperature during January–March on population density showed high precipitation with low temperature increased population density, while high precipitation with high temperature reduced in all regions (Fig. 4).

## Discussion

There were large fluctuations in both the population size of *S. mosellana* and climate factors during 1984–2013 in central China. We explored key climate factors associated with the field population density of *S. mosellana*. At the month scale, abundance is related to precipitation and the interaction between precipitation and temperature. At the annual scale, precipitation from January to March is a particularly important predictor.

*S. mosellana* diapause in the soil as larvae from summer to the following spring. Diapause is broken in response to rising soil temperatures, after which larvae enter a moisture-sensitive period. Most larvae developed when the soil moisture reached 17.5%, whereas most larvae went into an extended diapause for another year when the soil moisture was <12%^14^. Shanower (2005) highlighted the key role of soil moisture in enabling larvae to leave their cocoon^16^. Soil moisture also is the main factor limiting pupation and successful adult emergence^13,17,18^. Therefore, soil moisture levels during moisture-sensitive period is predicted to be a key factor affecting the yearly population abundance of *S. mosellana*. In central China, the soil moisture of wheat fields before April is determined by natural rainfall. The total precipitation during January–March in central China was greater in 1989 than in other years, which resulted in *S. mosellana* population outbreaks. On the contrary, the population size was very small if total precipitation during January–March was <20 mm. This result is consistent with the previous report, when precipitation exceeded 20 mm in May or early June in western Canada, the larvae terminated diapause, left their cocoons and pupated in several days, or most larvae remained dormant until the following year^18^.

Temperature may impact insects in many ways, directly via physiological or behavioral changes, or indirectly by influencing plant–insect interactions^19^. Within a certain range, increased temperature tends to have positive effects on insects^20^, especially multivoltine insects in temperate climate zones. However, our results indicate that the field population dynamics of *S. mosellana* was not directly determined by temperature. *S. mosellana* larvae pupate and emerge within the soil, and soil could therefore buffer these stages from the direct impacts of extreme temperatures. Similar results were also reported in other species such as parasitoids^21^, indicating that parasitoid development can be independent of the macroenvironment, and changes in temperatures are less likely to alter the dynamics of the host–parasitoid system^22^. The change of soil temperature lags behind that of air temperature, which may influence the population dynamics of *S. mosellana*. This requires further research as no data on soil temperature were available in the current study.

Many univoltine insect herbivores evolved their life history to synchronize their larval stages with the appearance of target host organs, and this synchronization determines the quality and quantity of available food resources and the population size of herbivores^23^. The life span of *S. mosellana* adults is usually a few days, and the damage occurs only if adult emergence coincides with the sensitive stage of wheat (ear emergence through to flowering). Therefore, the synchronization influences *S. mosellana* more severely than other insects. Climate changes may disturb the synchronization of herbivores with their host^23^. In this study, *S. mosellana* population size was significantly negative effected by interaction between temperature and precipitation, which showed that high precipitation with low temperature in spring also reduced the population density. The temperature requirement for *S. mosellana* adult emergence is different from that for wheat ear emergence; a cold spring may the later emergence of *S. mosellana* but may speed the ear emergence of wheat. In addition, the synchronization is affected by wheat variety, because the growth period will vary among wheat varieties^24^.

## Conclusions

Many studies and reviews report the effects of climate factors on multivoltine insects^2,5^, but there are fewer studies of climate impacts on univoltine insects. *S. mosellana* has one generation per year and spends more than 9 months in the soil as a diapause larva in a cocoon. The feeding-stage larva lives in a relatively confined space (wheat glume). Our results indicate that the population dynamics of *S. mosellana* were significantly related to precipitation during January–March in central China. Soil water evaporation is low during January–March in central China due to the low ambient temperatures^25^, so the soil moisture in late March and April was highly correlated to precipitation level during January–March. The soil moisture in late March and April could be a key factor determining the population dynamics of *S. mosellana* in central China. The low temperature in spring is likely to indirectly influence population dynamics by disturbing the synchronization of *S. mosellana* with wheat. These results provide basic information that will help in forecasting population levels and in developing a sound pest management strategy for *S. mosellana*.

## Methods

### Study region

**Figure 5:**
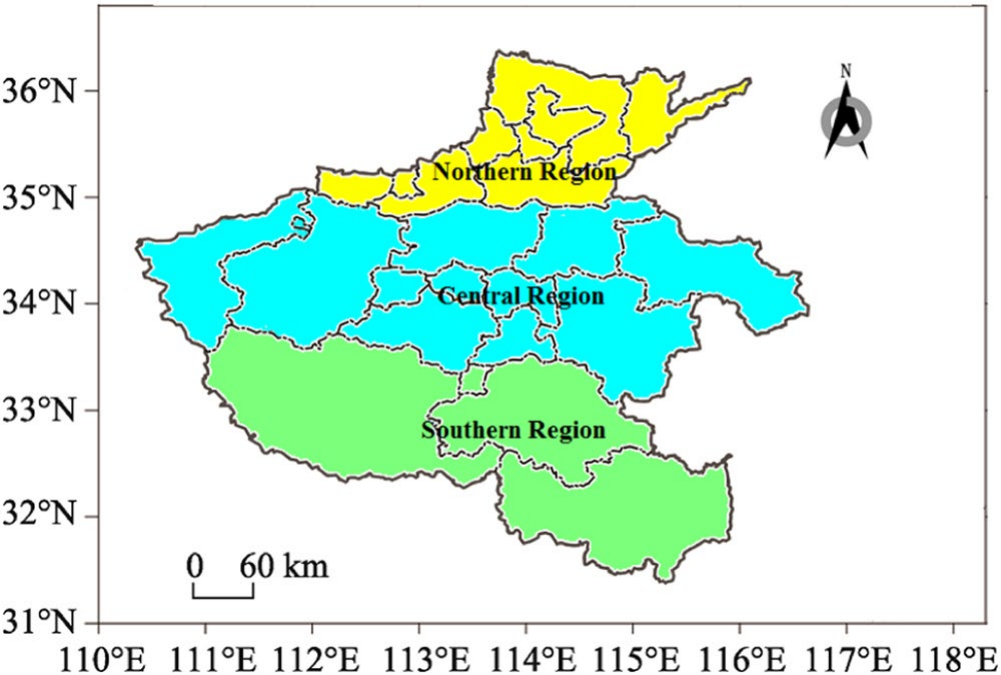
The Henan Province study region in central China. Data on the temperature, precipitation, and population densities of *S. mosellana* from 1984 to 2013 were collected in the southern, central, and northern regions.

Research was conducted in Henan province (31°23′–36°22′N, 110°21′–116°39′E, 167,000 km^2^), located in the central part of China. It is the main agricultural province and the largest winter wheat producer in China, producing 25% of the national wheat output. The climate in Henan province is continental monsoon and varies from the subtropical zone in the southern area to the temperate zone in the northern area. The mean annual temperature in Henan province ranges from 15.7 °C in the southern areas to 9.5 °C in the northern areas. The annual mean precipitation ranges from 533 to 1,380 mm, with the majority of rain fall occurring in summer. In this study, Henan province was divided into southern research regions (31°23′–33°50′N, 63,000 km^2^), which are located in the Huaihe river valley and belong to a subtropical humid monsoon climate with abundant rainfall and sunshine; northern research regions (35°00′–36°22′N, 26,000 km^2^), which are located north of the yellow river and belong to a warm temperate semi-humid and semi-arid continental monsoon climate; and central research regions (33°50′–35°00′N, 78,000 km^2^), which belong to a transition zone (Fig. 5). The mean annual temperature is 15.1, 14.6, and 13.6 °C in the southern, central, and northern regions, respectively. The mean annual precipitation levels are 806, 634, and 528 mm in the southern, central, and northern regions, respectively.

### Data collection

The data on annual cocoon densities of *S. mosellana* in the soil in three research regions from 1984 to 2013 were collected by the Plant Protection and Plant Quarantine Station of Henan province. Cocoon densities of *S. mosellana* in the soil were sampled during 1984–2013 by extracting soil samples in randomly selected wheat fields in which *S. mosellana* infestations occurred. Three soil samples (20 × 20 × 15 cm each) per field were bulked to represent each site. There are 88 counties in Henan province, and more than 10 sites were sampled in each county every year (>200 sites each region each year). Soil samples were taken along transects starting 15 m from the edge of the field, with samples per transect spaced 10–15 m apart. Cocoons were separated from soil samples by wet sieving, and collected with forceps and placed in alcohol for examination and counting. The mean density on all sites and soil samples represented the population density in a region.

Climate factor data (temperature and precipitation) were obtained from China Meteorological Data Sharing Service System, China Meteorological Administration (http://cdc.nmic.cn/home.do/). The monthly and annual climate data, mean temperature and total precipitation during January–March of each year were used to analyze the association with the population dynamics of *S. mosellana* in the three regions during 1984–2013.

### Data analysis

Data were evaluated for normality using the Shapiro–Wilk test prior to performing the analyses. One-way analysis of variance was used to compare the differences of *S. mosellana* population density, temperature, and precipitation among years using SPSS statistics software, version 22 (IBM Corp., Armonk, NY, US). The contribution of monthly precipitation and mean temperature on the population density of *S. mosellana* in the three regions of Henan province during 1984–2013 was evaluated using Generalized linear mixed effects models (GLMM) in package glmmADMB in R Statistical Software 2.13.1, using ‘temperature’, ‘precipitation’ as independent variables, ‘year’ as random variables, and Poisson error distributions. Post-diapause larval development and adult emergence period are moisture- and temperature-sensitive during late March and April in central China, so the temperature and precipitation during January to March were assumed the key factors determining the population dynamics of *S. mosellana*. To test this hypothesis, We used the generalized linear model (GLM) software package, relating the density of *S. mosellana* to temperature and precipitation during January to March.
